# Integration of breast cancer prevention and early detection into cancer palliative care model

**DOI:** 10.1371/journal.pone.0212806

**Published:** 2019-03-20

**Authors:** Adwoa Bemah Bonsu, Busisiwe Purity Ncama

**Affiliations:** 1 Discipline of Nursing, School of Nursing and Public Health, University of KwaZulu-Natal, Durban, South Africa; 2 Department of Nursing, Faculty of Allied Health Sciences, College of Health Sciences, Kwame Nkrumah University of Science and Technology, Kumasi, Ghana; Navrongo Health Research Centre, GHANA

## Abstract

**Background:**

Breast cancer is common among Ghanaian women. Late stage presentation has been credited to knowledge deficit and lack of breast cancer prevention and early detection services for women.

**Objective:**

This study aimed to develop a model to facilitate the integration of breast cancer prevention and early detection into cancer palliative care

**Method:**

This study used synthesized concepts emerging from a single case study research. The case was a tertiary health care facility, embedded with sub-units of analysis. Mixed-method approach was used to collect data from 102 participants. The study examined the experiences and views of the participants on breast cancer and screening pathways in Ghana. Thematic analysis and descriptive statistics ware used to analyze the qualitative and quantitative data respectively. This was followed with a cross-case analysis across the sub-units of analysis. A theory development approach was further used towards the development of a model, following three steps: concept analysis, statement synthesis and theory synthesis.

**Results:**

Six key concepts synthesized from the data were used to develop the model: initiate and sustain breast cancer prevention and early detection program, collaboration of health professionals, patients, families and micro-communities, conducive environment of the health care facility and needed resources, actions, services, and lastly diffusing innovation into the community through agents.

**Conclusion:**

A model has been developed based on the experiences shared by women diagnosed with advanced breast cancer, their first degree relatives, micro-communities as well as clinicians working in a palliative care setting. This model will aid clinicians to provide breast cancer education, teach breast self-examination and offer clinical breast examination to families and micro-communities of advanced breast cancer patients receiving supportive care in a resource-limited setting.

## Introduction

Earlier researchers have drawn attention on the problem of increasing and disproportionate burden of breast cancer (BC) in lower-and middle-income-countries (LMICs) in relation to the adoption of contextual proven interventions by countries [1–5]. Ghana is among the sub-Saharan countries in which BC is considered as a public health problem due to the increasing incidence coupled with advanced stage presentation and high mortality rate. The burden is further increased with the absence of a BC control and prevention plan [6–8]. Hence, the need to promote early detection to influence the <25% of 5years overall survival rate of the disease has been frequently highlighted [2, 9].

Although a relative significant improvement has been seen in the diagnosis and treatment of BC for detecting and treating new cases in recent years, despite deficiencies reported in prior studies, there are common interventions to influence early detection and prognostic outcomes that need to be developed and effectively integrated into the health system in response to the evolving nature of the disease [2, 10]. Ghana is striving towards incidence reduction, early detection and improving survival outcomes of BC as per Breast Health Global Initiative (BGHI) and World Health Organization (WHO) targets for achieving early detection of the disease globally, especially, in LMIC, where the impact of BC is greater [1, 3]. For a country to achieve early detection of BC and improve survival outcomes, health professionals need to be at the frontline to coordinate awareness and early detection services through an appropriate referral network for timely diagnosis and prompt treatment [11]. A model is required to facilitate the integration of breast health services into clinical practice. Palliative care (PC) for BC offers supportive care to patients living with advanced breast cancer (ABC) and their families. Hence, the clinic presents an opportunity for health professionals to initiate and sustain a prevention and early detection services for women who are exposed to the disease and experiencing it in the family. This study describes the development of a model to facilitate such a purpose. The model was developed based on data collected from stakeholders involved in BC including clinicians, patients, their family members and micro-communities.

### Aim of the study

To develop a model for the integration of BC prevention and early detection into cancer PC

#### Specific objectives

The study’s specific objectives were developed based on the processes outlined by Chinn and Kramer [12]

1. To select the concepts of the model from the study findings
2. To describe and define the key concepts identified
3. To develop the relationship statements among the concepts
4. To define the structure and process of the proposed model

## Methodology

### Study setting

The main study was carried out at the Komfo Anokye Teaching Hospital (KATH) in Kumasi. The palliative care clinic of the hospital served as the recruitment outlet for the study. Kumasi is the second largest national city and attracts Ghanaians from almost all regions of the country as well as other nationals from neighboring countries such as the ‘La Cote D’Ivoire’, and Burkina Faso. This suggests that the family mix in the hospital in this cosmopolitan city ensured varying rich and in-depth information in the data generated. KATH was purposefully selected for this study based on the increasing prevalence and incidence of BC coupled with late presentations of women with the disease. Data project that, incidence rate and advanced stage presentation of BC in Kumasi, the Ashanti region seems higher [6, 7, 13, 14] than other parts of Ghana [15–17]. KATH is a tertiary hospital within the structure of the Ministry of Health (MOH) of Ghana [18], and it is a large cancer referral center with a palliative care clinic, serving the Southern part of Ghana [6, 10, 19].

### Study design

As part of a large project, this study aimed to develop a model using constructs identified from an exploratory single case study which had embedded sub-units of analysis [20, 21]. The sub-units of analysis were: ABC patients, their first degree relatives, micro-communities, and clinicians working in a palliative care setting. The study concentrated on the stakeholders (herein referred to as sub-units of analysis) specific meaning of importance regarding their realities on BC prevention and early detection services [20]. This allowed an exploration of the phenomenon in an open, non-leading approach, which is the suitable way when conducting a theory-generation study [12]. Further, the three steps process for model development proposed by Chinn and Kramer were employed for the model herein reported [12, 22]: (1) concept analysis, (2) relationships statement and lastly (3) description of the model.

### Study sample

The case in this study was KATH. A total of 102 participants were involved in the study. These were women diagnosed with ABC, their first-degree relatives (sister, daughter and mother), micro-communities (non-blood relations; friend, co-tenant, church member and work colleague), and experts in a palliative care clinic. Table 1 below presents a summary of the sample size of the study participants:

**Table 1:** Summary of study participants.

Participants	Sample size
Patients with advanced breast cancer	11
First-degree relatives	17
Micro-communities	67
Clinician • Palliative care surgeon (1) • Palliative care Physician (1) • Radiation Oncologist (1) • Palliative care nurse specialist (1) • Medical Officers (2) • Palliative care resident (1)	7
**Total**	102

The socio-demographics data of the ABC patients are described in our previous study [23]. However, that of the first degree relatives, micro-communities and clinicians are shown in S1, S2 and S3 Tables respectively.

### Sampling techniques

A non-probability purposive sampling strategy was used to intentionally select the case and the sub-units of analysis (key stakeholders) to share their experiences on the phenomenon under study [20]. Eligibility was based on their involvement with BC and its related practices. Evidence captured from the various stakeholders yielded constructs for the model development.

### Data generation procedure

Based on the objectives of the study, qualitative and quantitative data were collected from different sources using interviews and questionnaires to triangulate and validate findings. This aided in the generation of rich and in-depth data which provided the concepts for the development of the model [21, 24]. Hence, the model was grounded on human interactions and the experiences of relevant stakeholders involved in the phenomenon of interest [25]. The languages for the data collection were English and ‘Twi’ (local language). The lead author, (ABB), a qualitative researcher who speaks and writes both ‘Twi’ and English languages and also has a clinical experience in oncology practice conducted all the interviews. ABB does not work at the palliative care clinic, therefore, she had no direct impact on the study participants and the site. Field notes were recorded during the interviews to include observable non-verbal cues of the participants and the researcher’s reflexibilities. Trained research assistants helped with the collection of the quantitative data.

#### Data analysis

The study was based on the findings of a study that explored and described the experiences of ABC patients, their first degree-relatives, and clinical experts in palliative care around BC and its related practices in their context. Based on the language of the interviews, transcriptions of all interviews were done either verbatim or based on the meaning of stakeholders’ comments [26]. The phenomenon was also examined among micro-communities of advanced breast cancer patients. Qualitative data for each sub-unit of analysis was analyzed concurrently with data collection following the techniques of thematic analysis and cross case analysis as described by Miles and Huberman [27] and Yin [21] respectively. Following the process of thematic analysis, each transcript was read repeatedly to make sense and meaning of the data and also assisted the researcher in generating findings for each of the sub-units of analysis [28]. This aided the researcher to identify patterns and generate relationships among participants’ thoughts by thematic coding, while comparing and contrasting. For Collis and Hussey [29], during thematic analysis, thematic coding limits researcher’s bias and ensures that codes and categories are grounded and inductively developed from the empirical data generated. As described by Miles and Huberman [30], a cross-case analysis was conducted through the search of similarities and differences across embedded sub-units of analysis. The themes were grouped into categories based on their relationships and patterns. This was an iterative process, as new themes emerged during the coding process; others were refined, deleted or merged in the process. The quantitative data was analyzed using descriptive statistics and merged with the qualitative data. This was followed by a cross-case analysis [21]. The cross-case analysis facilitated comparison of the four embedded sub-units of analysis using key features to identify similarities and differences across the units of data. Checklist matrix and case dynamic tables were developed to enable the cross-case analysis [30]. This assisted in the concept analysis across the sub-units of analysis, involving identification, definition and lastly, classification of the concepts identified based on their relations [12]. Reflective field notes recorded during the data collection were also used to give context to the data and substantiate the findings. The concept analysis was aimed at describing the experiences and perception of ABC patients, their first degree relatives, micro-communities and clinical experts in palliative care on BC prevention and early detection measures.

### Rigor

Credibility and authenticity of the findings was achieved through prolonged interaction with study participants during each interview section, triangulation of data using within and cross-case analysis and member checking to validate the findings [31]. Detailed description of the study’s methodological processes ensured the potential applicability and replication of the study by future researchers.

### Ethical approval

The study was approved by the Committee on Human Research, Publication and Ethics, Kwame Nkrumah University of Science and Technology and KATH, Ghana (Ref: CHRPE/AP/546/17 & CHRPE/AP/554/17) and Biomedical Research Ethics Committee, University of KwaZulu-Natal, South Africa (Ref: BE549/17). KATH permitted the study at the hospital (REG. NO: RD/CR17 /251). All participants gave their consent through writing before data collection.

### Theory underpinning the model

The Health **B**elief Model (HBM) was applied to the study [32]. The model has been identified as one of the approaches used to assess individual’s anxieties and fear about their health, their ability to follow health advices and how they relate with health professionals within the health care facility [33, 34]. Two significant components have been identified to affect the likelihood of a person choosing a recommended behavior in the model. These are knowledge and belief about BC susceptibility and severity. However, availability, affordability and accessibility to breast health services as well as stakeholders attitude are perceived as some possible barriers that may impede the uptake of BC prevention and early detection services. It is hoped that an increased understanding of the benefit of early detection and availability of essential resources, in the context of a conducive environment may increase high BC screening rate, leading to early detection of BC. The study also drew from the diffusion of innovation (DOI) behavioral theory by Rogers [35] which offered the ground to adopt methods for the quick dissemination and uptake of new program in BC prevention and early detection into communities through patients, families and micro-communities. The study is further founded on the recommendations by WHO[36] and BHIG [1] which offer the basis for every country to develop a cost effective and contextual BC prevention and early detection model and integrate it into health care systems to facilitate early detection of the disease among women.

### Synthesis of theory

The model was developed using identified and defined concepts, followed by the development of relational statements which linked the identified concepts. Conceptual model and graphical representation is used to clearly illustrate relationships that exist within and among concepts [37]. The ensuing sections describes the steps followed to develop the model.

### Step one: Concept analysis

A concept is described as a mental representation of a phenomenon or global views and are made of features that are abstract from reality [12, 37]. The concepts used to develop the model in this study were analyzed in a two-phase process; (1) identification of main and related concepts, and (2) classification and definition of identified concepts.

#### Phase one: Identification of main concepts

In this study, primary concepts for the model were identified from the results of the cross-case analysis of all the data generated [24]. Thirty-five (35) concepts were identified in total and were further synthesized by analyzing their similarities and differences across, resulting in the final deductive generation of six (6) main concepts. These were used to develop the model. The main concepts are: S4 Table presents the concepts in detail ***(please insert S4 Table here).***

1. Initiate and sustain BC prevention and early detection program.
2. Collaboration between health professionals, patients, families, and micro-communities.
3. Conducive environment of the health care facility and needed resources.
4. Actions.
5. Services.
6. Diffusing innovation into community through agents.

#### Phase two: Classification and definition of main concepts

The concepts were classified in accordance with Dickoff el al [38] list of survey; and defined using a dictionary contextualized coupled with subject definitions. Six questions relating to the aspects of the activity were employed to survey activity as follows:

1. ***Agent*:** Health professionals are the agents of this model. As professionals, they are obliged by their professional philosophy to deliver quality health care services to patients and their families and implement interventions aimed at preventing diseases and promoting the health of patients, family and the community.
2. ***Recipient*:** The patients, their families and micro-communities are the recipients of the model and are expected to follow the advice given by the agent. Further, they are significant stakeholders of the model, hence, their collaboration with the agents to achieve a common aim is crucial. The model will also be extended to other women in the communities through the recipients as agents of change.
3. ***Context*:** The context is a PC clinic in a tertiary healthcare setting. This will guide the agents to integrate BC prevention and early detection services into their clinical practice.
4. ***Procedure*:** As described by Bertram and colleagues [39], this study revealed that integration is a phase-related-process. Hence, specific conditions and activities are needed at each phase. The process of integration requires involvement of the agent context and the recipient.
5. ***Dynamics*:** Breast cancer prevention and early detection services are dynamic and mutual interactive process between the agent, the context and the recipient. The context through which the agents provides and delivers the service plays a significant role during implementation of the program. The agent establishes an enabling working relationship with the recipient. This interaction creates a path through which dynamic processes for effective BC prevention and early detection interventions are facilitated.
6. ***Purpose (Terminus)*:** To achieve an effective integration of a BC prevention and early detection program, through collaborating stakeholders towards the realization of a common goal.

### Step two: Definition of main concepts with their relationship statements

Relational statements were developed through the identification of the relationship between the concepts identified in step one [37]. Dictionary and subject-specific definitions were used to define the main concepts. The definitions are contextualized in accordance with the study’s objectives to enhance meaning to the various parts within model.

1. **Initiate and sustain BC prevention and early detection program:** This main concept has eight related concepts as presented in S4 Table (supplementary file). The dictionary definition for the concept is as follows:
  - **Initiate:** Refer to begin, set going or originate [40].
  - **Sustain:** Means cause to continue for an extended period or without interruption and to strengthen or support physically or mentally [41].
  - **Breast:** Serves as the mammary gland, with a major function of nutrition for infants. It also has social and sexual features [42].
  - **Cancer:** Refers to the uncontrollable growth of abnormal cells in the body [43].
  - **Prevention:** Is the act or practice of stopping something bad from happening or arising [44].
  - **Early detection:** Is the process of noticing or discovering something [44].
  - **Program:** a planned, coordinated group of activities, procedures or actions often for a specific purpose [41].
  - **Subject and contextual definition:** In this study, the concept denotes a coordinated group of breast health activities to start at a PC clinic and maintained for patients, their family and micro-communities. These group of activities will be offered by health professionals at the PC clinic to facilitate the prevention or early detection of breast abnormality.
  - **Relationship statement:** Initiation of a program, in this context will be preceded by planning, which will prepare the agents for the program. This will help the health professionals to understand the program and clarify how the program will be initiated, manage and sustained. Health professionals will be aware of their roles as agents of the program and how to execute their roles. Planning will involve engagement between all relevant stakeholders of the program. This will help to address exiting challenges that may impede on the program and further provide the resources required for the program to start.
2. **Collaboration between health professional, patients, families, and micro-communities:** This concept has 4 related concepts (S4 Table). The dictionary definition of concept is presented below.
  - **Collaboration:** refers to the situation of two or more people working together to create or achieve the same thing [34].
  - **Health professionals:** Someone who works in the medical profession, for example, a doctor or nurse.
  - **Patient**: a person who is receiving medical care, or who is cared for by a doctor or specialist when necessary.
  - **Family:** Any group of persons either related by blood, as, parents, children, uncle, aunts, cousins or generally not blood relations but who share common attitudes, interest and goals [30].
  - **Subject definition of family**: Is an institution in the center of the Ghanaian society, sustained through a series of kinship network, marriage, and other social relations, and, it is acknowledged as the bedrock of all social life. The family is the main source of social security during illness (emotional, physical and financial) and provides caregiving support to its members. Other significant social relations such as friends are also considered as family [35].
  - **Micro**- Very small or at the lowest level [41].
  - **Community**: People living in one area or people who are considered as a unit because of their condition of sharing or having certain attitude, common interest, social, group or nationality or background [41].
  - **Subject definition of micro-community:** This denotes a unit of people relating with the patient and share common interest as a social group with patients, for example, friends, co-tenants, church members, and working colleagues.
    - **Subject and contextual definition:** in the context of the study, collaboration with health professionals, patients, families, and micro-communities denotes all the stakeholders working together as a team with a responsibility and interest towards breast cancer prevention and early detection.
    - **Relationship statement**: Patients, families, and micro-communities are to accept and actively contribute during the process of the program development and its implementation. The interaction and engagement between the agents and the recipients of the program facilitates team work and partnership. This is perceived by as beneficial for the BC prevention and early detection program.
3. **Conducive environment of the health care facility and needed resources:** This concept has eight related concepts. The ensuing paragraphs presents the dictionary definition of concepts.
  - The Online Cambridge English Dictionary [44] defines ***conducive*** as providing the right conditions for something good to happen or exist.
  - **Environment:** Is the physical, or social and cultural surroundings that influences the life of an individual or community [40].
  - **Conducive environment:** Hence, refers to the physical, or social and cultural surroundings that influence the life of an individual or community for something good to happen or exist.
  - **Subject definition of conducive environment:** conducive environment is a place where people live and work; interact and build beneficial relationships. It creates the opportunity for people to share and acquire knowledge, new skills and talents.
  - **Health care**: Defined as the organized provision of medical care to individuals or a community [41].
  - **Facility**: Refers to equipment, room or building that is used for a particular activity or purpose [44].
  - **Subject definition of healthcare facility**: In this study, it denotes the organized provision of supportive care services to patients diagnosed with advanced breast cancer, their families and micro-communities at a PC clinic of a tertiary health institution.
  - **Resources:** Refers to a stock or supply of money, materials, staff, and other asserts that can be drawn on by a person or organization in order to function effectively [41].
  - **Subject and contextual definition:** Conducive environment of the health care facility and needed resources refers to the conducive PC clinic within a health care facility, staff with competent health professionals and stock with all the supplies needed to provide BC prevention and early detection for women.
  - **Relationship statement:** Needed resources for effective integration of BC prevention and early detection program include physical space, adequate staff strength, BC and BSE leaflets (brochures) and policy. Further, management commitment and support, attitude of health professionals and self-motivated health professionals are additional elements that are needed to make the environment conducive for the integration of the program. These resources are vital to assist health professionals work effectively within their scope. This will also foster the provision of quality breast health series to patient’s family and micro-community at the palliative care clinic.
4. **Actions:** This concept has two related concepts (S4 Table). The concept is defined below:
  - **Actions:** Defined as to begin working to make an idea or a plan happen or be successful [44].
  - **Subject and contextual definition:** Actions refers to all the activities that will be implemented to the BC prevention and early detection program successful. These include advocacy among health professionals to build their competency on BC, education, counseling and breast examination. The health professionals will incorporate the acquired skills and knowledge gained to counsel and educate women on BC, teach BSE and offer CBE for women during their clinical practice.
  - **Relationship statements:** Actions will build the competency of the health professionals and assist them to deliver breast health services and counseling confidently and efficiently to women during their clinical practice at the palliative care clinic.
5. **Services:** This concept has 5 related concepts and it is defined below.
  - **Services:** denotes a government system or private organization that is responsible for a particular type of activity, or for providing a particular thing that people need [44]
  - **Subject and contextual definition:** in this study, services refer to provision of BC prevention and early detection activities by to women.
  - **Relationship statements:** Health professionals will educate women on BC, teach BSE, offer CBE, offer counseling before and after CBE as means of providing BC prevention and early detection services to the women. Also, as means of follow-up care, the health professionals will encourage the families and micro-communities to practice monthly BSE and visit the PC clinic periodically for CBE. In addition, the women will be urged to be confident and discuss any abnormal findings or suspicions with their health professionals for prompt evaluation [36, 45].
6. **Diffusing innovation into the community through agents:** This concept has 4 related concepts and it is defined as below.
  - **Diffusing:** Refers to spreading or disseminating or broadcasting something over a wide area in many direction or between a large number of people [40].
  - **Innovation:** Means the development of a new idea or method, design, product or the use of new ideas and methods [41].
  - **Agent:** A person who takes an active role to produce a particular effect of change [41].
  - **Subject definition of agent**: In the context of this study, agents for the communication will be health professionals, patients, families and the micro-communities. The health professionals will actively communicate the program to the recipients and offer the services of the program to the recipients at the PC clinic. Patients, families and the micro-community will take an active role in carrying message of the innovation to other women in their communities to increase the uptake of the program.
  - **Subject and contextual definition of concept:** in this study, diffusing innovation into the community through agents refers to patients, families and micro-communities, acting within the community (social system) as agents of change, spreading information about BC prevention and early detection practices to other women within their communities (households, vicinity, workplace, church and other social centers). Trying to influence other women’s beliefs and behaviors around BC in a direction deemed desirable by the health professionals. They will share acquired information with other women within their communities and direct them to access the BC prevention and early detection services at the PC clinic.
  - **Relationship statement:** The health professionals will interact with the patients, families and the micro-communities through communication and convey the knowledge they have acquired on BC to them. They will engage in conversation with the recipients on their breast health and encourage them to share and convey the innovation to other women within their communities, as means of acting as active agents of the program. They will encourage the recipients to direct other women in their communities to the PC clinic to access the program. Feedback from the recipients on the performance of the program will be used to evaluate the effectiveness of the program.

**Fig 1:**
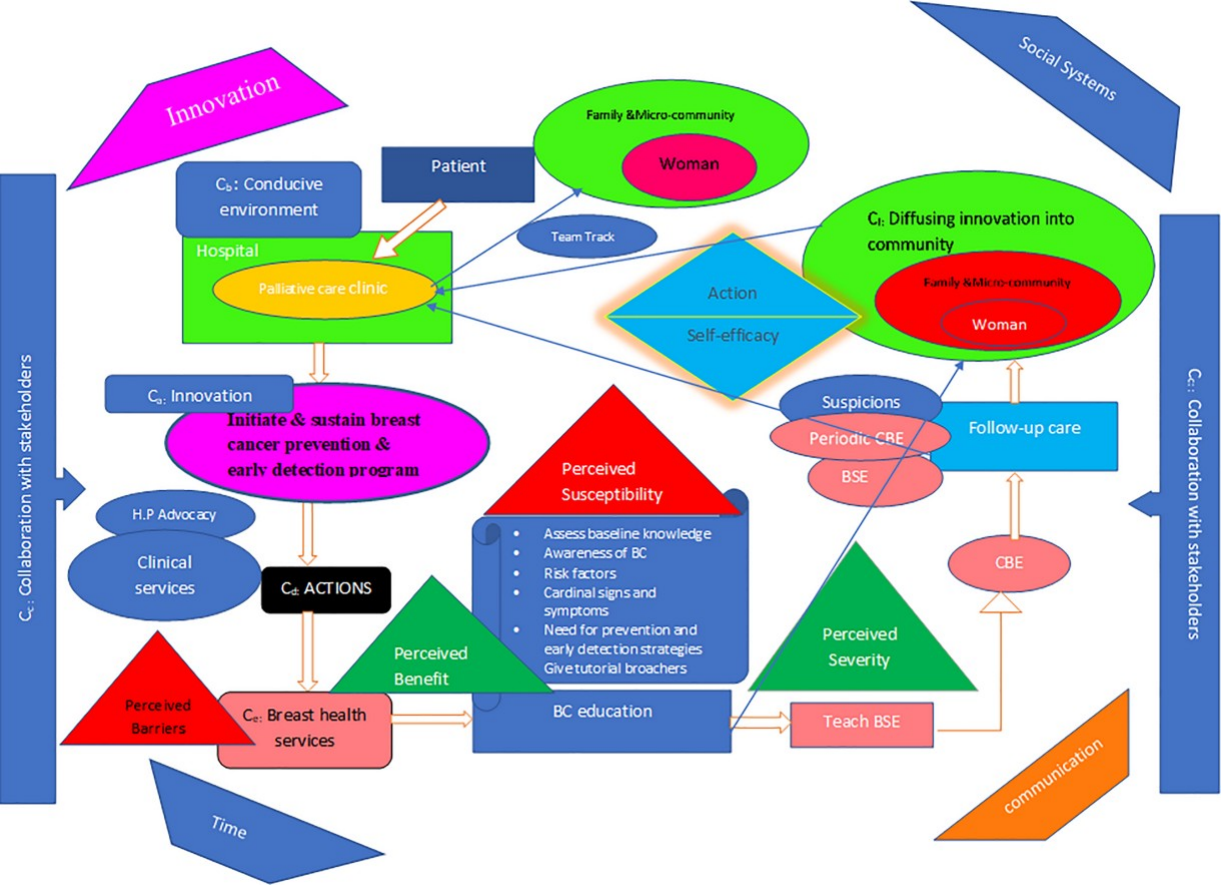
Integration of prevention into cancer palliative care model.

The model is illustrated in Fig 1 below.

### Step three: Description of the model

#### Assumption of the model

The model is grounded on the philosophical assumptions of the HBM [32] and further drew on the Diffusion of Innovation theory (DOI) [35] as afore-mentioned. The family members and micro-communities of ABC patients will believe that:

- They are likely to develop BC in the future (perceived susceptibility).
- Engaging in prevention and early detection services such as health education and breast screening will be effective (perceived benefit)
- Breast cancer is severe and a threat to their health, hence, adhering to advice given by the health professionals at the palliative care clinic on BSE and CBE will be beneficial to their well-being (perceived severity)
- Barriers such as travel cost to the clinic, time spent at the clinic, attitude of health professionals may impede on the use of the service, however, they will manage to decrease their perceived barriers (perceived barriers).
- The women will be encouraged to likely utilize the model by accessing BC education and screening at the PC clinic (action)
- The women will be confident in their ability to do monthly BSE and periodic CBE and discuss concerns with their health professionals for prompt actions (self-efficacy).
- The women will speed up the spread and implementation of new ideas in BC prevention and early detection program to other women in their communities.

#### Structure of the model

Concepts are well understood and perceived as real when they are presented in a structural form to illustrate how they are interrelated [24, 37]. The structure of this model is grounded in the findings generated from a larger study and shows how the main concepts are interrelated. Thus, the recommendations made by the participants of the study were used to develop the structure of the model. The structure of the model will be described briefly under sub-themes using the concepts to aid in comprehensive understanding of the model. To ensure clarity, letter ‘C’ has been used to label the ‘concept’ and subscript lowercase letters (a, b, c, d, e and f.) denote the order of the concepts. For instance, C_a_ denotes ‘initiate and sustain BC prevention and early detection program’, hence the first concept (concept 1). The numbering of the concepts is to simplify the description of the model structure, but it is not of any significant importance. Although the process of promoting BC prevention and early detection is sequential, it is iterative.

#### Initiate and sustain BC prevention and early detection program (C_a_)

The model is an innovation that requires planning. Leaders of the hospital are the driving force for the initiation and integration of a program, hence, their support is paramount [46]. They will provide directives on how the model should enroll by developing a time-bound plan for the integration in reference to the resources available and the context for the integration [39]. The leaders will communicate the vision and aims the program hopes to achieve to the agents and clarify the roles of the various players of the program [46]. The readiness of the agents promotes the effectives of the implementation. To sustain the program, the recipients’ acceptance and participation in the program is paramount.

#### A conducive environment of the health facility and needed resources (C_b_)

The environment for the integration will be the PC clinic of a tertiary health care facility. This environment can either support or impede on the program [47, 48]. Enabling environment is required for the implementation of the services. Support from the leaders of the health institution will aid health professionals to be proactive in developing locally compactible ideas that will facilitate program integration and its sustenance. There is the need for the management of the health institution to formalize the model to aid its integration into the PC clinic of the hospital.

Physical, human, material and financial resources are needed for effective integration of the model. Physical space needs to be created at the PC clinic to enroll the program. Also, the staff strength of the clinic needs to be adequate and the required materials (brochure) and equipment needed for the work should also be provided. Due to the absence of BC protocol in Ghana, no national budget has been allocated for BC prevention and early detection programs in Ghana. Hence, financial support from individual donors, private organizations, religious organizations and NGOs will be required to sustain the program. Absence of resources may adversely affect the BC prevention and early detection services to be provided to the recipients. Previous authors have confirmed that lack of adequate resources impede on implementation of program [47].

#### Collaboration of health professionals, patients, families and micro-communities (C_c_)

The model needs collaboration support from its initial stage and continued throughout the period of implementation to enhance its sustainability. Teamwork among stakeholders is therefore seen as paramount to facilitate the integration of the prevention and early detection of a BC program for Ghanaian women. Acceptance and uptake of the program requires the involvement and engagement of the patients, families, and micro-communities [49]. They form integral part of the health system structure, hence, their constructive input will facilitate the integration of a contextual BC prevention and early detection program [47]. Further collaborative support from various private organizations and NGOs is also essential. Various collaborative assistance of importance includes advocacy and awareness creation on BC, equipment and financial support [45].

#### Actions (C_d_)

Actions depicts the various activities that will be involved in the BC prevention and early detection program.

**Health professionals advocacy**: Health professionals are the agents of the program; hence, their competency is very significant to the implementation of the program. To ensure the delivery of quality breast health services, the knowledge and skills of the agents on the disease, education, counselling and breast examination should be enhanced. Health professionals should hence, be trained in these regards. This will build the competency and the confidence of the health professionals for effective integration of the program.

**Clinical services:** Health professionals will incorporate the prevention and early detection services into their clinical practice. These clinical services will be delivered to families and micro-communities who accompany ABC patients referred to the PC clinic for supportive care.

#### Services (C_e_)

These are the interventions that will be provided for women in the BC prevention and early detection model.

**Education of women on breast cancer:** The baseline knowledge on BC and its practices of women who accompany ABC patients to the PC clinic should be assessed and built of from the known to the unknown. Education on BC should include modifiable and non-modifiable risk factors of the disease, cardinal and vague symptoms of the disease, measures relevant for symptom discovery, path to early detection, significance of early detection as well as late stage presentation to survival and treatment outcomes [45]. Women should also be educated on prevention measures and the need to modify their lifestyles to prevent the development of the disease. The beliefs and religion of the women should be acknowledged by health professionals during the education sessions, as these elements are significant aspect of the socio-cultural context of Ghana [36]. Language for the education should also be considered to enhance understanding Leaflets containing information on BC should be provided to the recipients to serve as a reminder. Recipients should be encouraged to express their concerns and ask questions on the education given to enhance understanding. Adequate and simple answers should be provided to clear all misconceptions. Recipients should also be assessed on the education given

**Teachings on BSE:** Early detection of BC in developing countries largely depends on women who practice regular BSE and self-refer [16]. Hence, from the model, woman should be given practical teachings on BSE to enhance their practicing skills. Women should further be taught the right time of the examination to maximize its effectiveness. Leaflets containing information on how to do BSE should be given to guide the recipients in their monthly practice.

**Breast screening:** Health providers should offer CBE to asymptomatic women who visits the clinic. This may be more opportunistic in nature, because, women visit the clinic with the ABC patients on referral and appointment basis. Hence, health professionals will take advantage of such visits and offer CBE to the family member or micro-community of the patient [11].

**Follow-up care and referral:** Women should be offered the opportunity to visit the clinic periodically for CBE. The women should be encouraged to do BSE monthly. Also, health professionals should urge women to be self-confident to report and discuss any breast-related concerns which may be discovered with their clinicians. Where necessary, the health professionals as the front-liners of the BC prevention and early detection program will coordinate through a referral network for timely diagnosis and prompt treatment, particularly, when a suspicion is identified [11].

#### Diffusing innovation into the community through agents (Cf)

The dissemination of the new idea on BC prevention and early detection into the community was considered significant in this model and it includes two processes. First, institutional-level communication to facilitate discussions among the multidisciplinary health teams about the existence of the program and the best way to work as a team to achieve effective integration of the program and maximize the recipients’ benefits. The patients, families and the micro-communities must also accept and practice what is communicated by the health professionals to achieve a positive outcome of the program.

The four squares appearing in the four corners of the model (Fig 1): innovation, time, communication and social system are the vital elements required across the main concepts of the model for effective integration and sustenance of the BC prevention and early detection program. These are the four elements of diffusion described by Rogers [35]. For Rogers [35], innovation is an idea and practice perceived as new by an individual or other unit of adoption. The adoption or rejection of the idea is credited to contributing factors as perceived by the individual as ***relative advantage*** (perceiving the idea as being better than preceding practices), ***compatibility*** (consistency of new idea with existing beliefs, values, norms, need and past experiences), ***complexity*** (perceiving the idea as either easy or difficult to understand and use), ***tri-ability*** (how the new idea can be tried before making an informed decision to adopt it definitely) and lastly, ***reinvention*** (ability of the individual to customized and change the idea to maximize its usage). The BC prevention and early detection program at the PC clinic for patients, families and micro-communities is a new idea which requires that all potential stakeholders buy in and take ownership through these five attributes. The model is clear with added value for BC prevention and early detection, which has been contextually developed using stakeholders constructs to enhance its compatibility with existing socio-cultural norms. The services in the program are simple and can be easily understood by users, it can be tried, and it has clearly visible beneficial outcomes from using it. Potential users can own the idea and customize it for effective implementation.

The ‘time’ element describes the innovation decision process using knowledge, persuasion, decision, implementation and confirmation [35]. Awareness about the existence of program, the benefit and detailed information on BC, its prevention and early detection and practices will be provided by the health professionals to the recipients during their clinical services. Due to the perceived susceptibility of the recipients, they may form a positive attitude towards the breast health services provided at the PC clinic, make a decision to adopt it consciously or unconsciously, practice the innovation and evaluate their previous innovation decisions [35]. It is acknowledged that the recipients may fall in any of the five groups of adopters, which include innovators, early adopters, early majority, late majority and lastly laggards. It is however hoped that the recipients may perceive the program as beneficial and may control potential barriers that may impede on the adoption of the BC prevention and early detection practices.

Communication is the third element of the diffusion of innovation theory [35]. The patients, family members and micro-communities are from a community and they share common norms, values and beliefs with other community members. Therefore, extension of the BC prevention and early detection services into the community is a significant aspect of this model. To optimize the uptake of the program and its sustainability, peer education (interpersonal communication) as described by Rogers [35] to speed the spread of the idea into the social system (community) should be adopted.

Health beliefs and disease representation are noted to influence health behaviors towards the disease [50]. This model therefore targeted the patients, their families and micro-communities to act as an effective agents of change by disseminating information about BC, its prevention measures, BSE and CBE practices to other women in their community and influencing norms and beliefs in their community [51]. The agents of change will be urged to encourage their community members to access the program at the PC clinic. This will be an effective tool to change the cultural narratives around BC in Ghana and correct misconceptions and prevailing maladaptive beliefs held by Ghanaian societies about BC. Previous studies that drew on this peer education approach in the context of cervical cancer demonstrated increased awareness and uptake of cervical cancer screening services by women [52, 53].

The fourth element of the DOI is the social system which determines the boundaries of diffusion [35]. Opinion leaders in the health care facility will influence other health teams within the internal social system (hospital setting) in an appropriate way to accelerate the program’s integration and substance. Patients, their families and micro-communities will work within their communities and try to influence the innovation-decision of other women in a desirable direction to suit the aim and interest of the BC prevention and early detection program.

The model will also fall on the positive health beliefs of Ghanaians that can facilitate awareness and early detection of BC to help achieve stage shifting of BC in Ghana. Consequently, the five years overall survival rate and prognostic outcome of BC will be improved among Ghanaian women. Finally, the recipients of the model will be encouraged to provide feedback on the program’s implementation to help assess the effectiveness of the program.

### Outcomes

The following outcomes are hoped for:

- Effective integration of the BC prevention and early detection program
- Increased skills and competency among health professionals in delivery of breast health services
- Delivery of quality breast health services to families and micro-communities of ABC patients at the PC clinic
- Increased participation of women and their communities in the BC prevention and early detection program
- Increased awareness and knowledge of BC and screening measures among women
- Increased efficacy among women in the practice of monthly BSE
- Increased self-confidence in women to discuss BC related concerns with health professionals
- Effective dissemination of the BC prevention and early detection ideas into communities through women as agents of change

## Limitation and strength

The model was not piloted to evaluate its effectiveness, as this was out of scope for this study. Further, participants were drawn out of one institution and this may have generalizability implications. Irrespective of the above limitations, the study proposed a contextual model as a framework to guide health professionals to integrate BC prevention and early detection into supportive care, the last phase of the cancer continuum. A proposed knowledge to shift the paradigm of PC from suffering to prevention. This is a unique strength of this study. To the best of our knowledge, this study is the first to report on a model integrating BC prevention and early detection into a cancer palliative care within the socio-cultural context of Ghana.

### Barriers to effective integration of the model

The focus of this model is to promote prevention and early detection of BC among women, and it is hoped to reduce advanced stage presentation of BC and improve survival and prognostic outcomes of the disease among women within limited- resource setting. However, the absence of some essential factors may impede the integration of the model. These include the following:

- Conducive environment
- Stakeholders collaboration
- Administrative support
- Formalized policy
- Human resource (increase staff strength)
- Physical space
- Communication value
- Advocacy among health professionals
- Material and equipment (brochures on BC and BSE)

### Monitoring and evaluation of the model

The researchers acknowledge that monitoring and evaluation will facilitate the application of the model as envisioned and it is effective. However, evaluation of the model was out of scope of this study. Hence, the authors propose that the model should be tested and evaluated by future researchers for its effectiveness. Further, monitoring will ensure that the model fits well into the interest of women to which it serves to benefit. BC outcome measures such as incidence, survival and death rates as well as stage of presentation can be used as performance indicators to monitor the program. Health professionals can also use the success of the model’s implementation to monitor and evaluate the program. The satisfaction and other feedback from the recipients of the program is also paramount in evaluating the model.

## Recommendation and suggestion for the model

The model illustrates a new framework for prevention and early detection of BC within an institutional-based care. It can be used to promote awareness and early detection of BC among women in Ghana and beyond. Although the model was developed within a PC setting, the authors recommend it to be adopted and adapted in other context within healthcare settings irrespective of the disease condition to facilitate prevention of several disease conditions. Guidelines for the implementation of the model is required. The authors recommend future research to test the model and use the findings to scientifically develop a guideline that will facilitate effective implementation of the model.

## Conclusion

The study described a model developed to facilitate an integration of BC prevention and early detection into cancer palliative care. The model will guide health professionals working in a palliative care clinic of a tertiary hospital to deliver BC prevention and early detection services among family members and micro-communities of women diagnosed with ABC. The study drew on the DOI theory by Rogers to facilitate the speed dissemination of the program into women’s communities, using the recipients as agents of change. The procedural steps of the model development were presented as well as measures employed to achieve rigor. Ethical considerations, limitations, strength as well as recommendations were described. The researcher hopes to evaluate and ascertain the effectiveness of the model in the future.

## Supporting information

S1 Table This is the S1 Table Participants profile (First-degree relatives). (DOCX)

S2 Table This is the S2 Table Participants profile (Micro-communities). (DOCX)

S3 Table This is the S3 Table Participants profile (Clinicians). (DOCX)

S4 Table This is the S4 Table Concepts identification and classification process. (DOCX)
