# Supplementary material for: Integration of breast cancer prevention and early detection into cancer palliative care model
Source: PLoS One. 2019 Mar 20;14(3):e0212806. doi: 10.1371/journal.pone.0212806 (PMC6426220; doi:10.1371/journal.pone.0212806)
Supplement: S1 Table — (DOCX) [file pone.0212806.s001.docx]

**S1 Table : Profile of first-degree relatives**

| **Pseudonyms** | **Age range**  **(years)** | **Educational background** | **Religion** | **Marital**  **Status** | **Number of children** | **Occupation** | **Prior family history of cancer** | **If yes, relation** | | **Type of cancer** | **Relation diagnosed** |
| --- | --- | --- | --- | --- | --- | --- | --- | --- | --- | --- | --- |
| Nancy | 41-50 | Primary | Christian | Divorced | 2 | Trader | Yes | Father & Grand parents | Cervix & prostate | | Sister |
| Tina | 31-40 | Secondary | Christian | Married | 2 | Public servant | No | - |  | | Mother |
| Anita | ≥61 | Middle | Christian | Married | 1 | Pensioner | No | - |  | | Sister |
| Ruth | 21-30 | Tertiary | Christian | Single | - | Student | Yes | Grand mother | Cervix | | Mother |
| Gladys | 31-40 | Tertiary | Christian | Single | - | Student | Yes | Grand mother | Cervix | | Mother |
| Joyce | 21-30 | Tertiary | Christian | Single | - | Student | No | - | - | | Mother |
| Jane | 21-30 | Secondary | Christian | Married | 2 | Trader | No | - | - | | Mother |
| Faith | 41-50 | Middle | Christian | Single | - | Self employed | No | - | - | | Mother |
| Grace | 41-50 | Middle | Christian | Married | 2 | Trader | No | - | - | | Mother |
| Hannah | 51-60 | Secondary | Christian | Married | 2 | Trader | Yes | Aunty | Breast | | Sister |
| Vero | 21-30 | Middle | Christian | Married | 2 | Trader | Yes | Grand mother | Breast | | Mother |
| Gina | 31-40 | Tertiary | Christian | Married | 3 | Teacher | No | - | - | | Mother |
| Ceci | 41-50 | Primary | Christian | Married | 9 | Trader | Yes | 2 Sisters | Breast | | Sister |
| Rose | 21-30 | Secondary | Christian | Single | - | Unemployed | Yes | 2 Aunties | Breast | | Mother |
| Betty | 31-40 | Middle | Christian | Married | 3 | Self employed | No | - | - | | Sister |
| Olivia | 51-60 | Tertiary | Christian | Married | 4 | Pension | No | - | - | | Sister |
| Mary | 31-40 | Middle | Christian | Single | 1 | Trader | No | - | - | | Sister |
