# Supplementary material for: Integration of breast cancer prevention and early detection into cancer palliative care model
Source: PLoS One. 2019 Mar 20;14(3):e0212806. doi: 10.1371/journal.pone.0212806 (PMC6426220; doi:10.1371/journal.pone.0212806)
Supplement: S2 Table — (DOCX) [file pone.0212806.s002.docx]

**S2 Table: Participants’ demographical characteristics (Micro-community)**

| Demographic data | Frequency (n=67) | Percent (%) |
| --- | --- | --- |
| Age group |  |  |
| 18-39 | 36 | 53.7 |
| 40-45 | 13 | 19.4 |
| 46-50 | 10 | 14.9 |
| 51-55 | 3 | 4.5 |
| 56-60 | 4 | 6.0 |
| 61-65 | 1 | 1.5 |
|  |  |  |
| Religion |  |  |
| Christian | 52 | 77.6 |
| Moslem  Traditional | 14  1 | 20.9  1.5 |
|  |  |  |
| Educational Background |  |  |
| Primary | 8 | 12.0 |
| Middle | 12 | 18.0 |
| Secondary | 12 | 18.0 |
| Tertiary | 27 | 40.0 |
| None | 8 | 12.0 |
|  |  |  |
| Occupation |  |  |
| Farming | 3 | 4.5 |
| Trading | 16 | 23.9 |
| Self-employed | 8 | 11.9 |
| House wife | 5 | 7.5 |
| Public servant | 14 | 20.9 |
| Unemployed | 9 | 13.4 |
| Other | 12 | 17.9 |
|  |  |  |
| Marital Status |  |  |
| Married | 30 | 44.8 |
| Single | 26 | 38.8 |
| Divorced | 4 | 6.0 |
| Separated | 2 | 3.0 |
| Widow | 5 | 7.4 |
|  |  |  |
| Parity |  |  |
| Yes | 38 | 56.7 |
| No | 29 | 43.3 |
|  |  |  |
| Family history of breast cancer |  |  |
| Yes | 16 | 23.88 |
| No | 51 | 76.12 |
