# Supplementary material for: Integration of breast cancer prevention and early detection into cancer palliative care model
Source: PLoS One. 2019 Mar 20;14(3):e0212806. doi: 10.1371/journal.pone.0212806 (PMC6426220; doi:10.1371/journal.pone.0212806)
Supplement: S3 Table — (DOCX) [file pone.0212806.s003.docx]

**S3 Table : Participants profile (Clinicians)**

| **Pseudonyms** | **Age group (Years)** | **Gender** | **Educational level** | **Profession** | **Specialty** | **Current grade** | **Palliative care team member** | **Directly involve in breast cancer care** | **Years of practice in palliative care & breast cancer** | **Current position in the hospital** |
| --- | --- | --- | --- | --- | --- | --- | --- | --- | --- | --- |
| Pat | 31-39 | Female | Ph.D. | MD | Palliative Surgery | Specialist | Yes | Yes | 6 | Palliative Surgeon |
| Kate | 40-50 | Female | MSc. | Nursing | Oncology and Palliative | Nurse specialist | Yes | Yes | 15 | Oncology and Palliative care nurse |
| Baba | 40-49 | Male | Ph.D. | MD | Radiation Oncology | Specialist | Yes | Yes | 8 | Radiation Oncologist |
| Agyapong | 31-39 | Male | Fellowship | MD | Palliative | Specialist | Yes | Yes | 3 | Palliative Care Specialist |
| Kofi | 31-39 | Male | Tertiary | MD | Palliative Oncology | Resident | Yes | Yes | 6 | Resident |
| Adizah | 31-39 | Female | Tertiary | MD | General practitioner | Senior Medical Officer | Yes | Yes | 4 | Senior Medical Officer |
| Akwasi | 31-39 | Male | Tertiary | MD | General practitioner | Senior Medical Officer | Yes | Yes | 5 | Senior Medical Officer |
