# Supplementary material for: Integration of breast cancer prevention and early detection into cancer palliative care model
Source: PLoS One. 2019 Mar 20;14(3):e0212806. doi: 10.1371/journal.pone.0212806 (PMC6426220; doi:10.1371/journal.pone.0212806)
Supplement: S4 Table — (DOCX) [file pone.0212806.s004.docx]

**Table 4: Concepts identification and classification process**

| **Model for breast cancer awareness and screening** | **Concluding statements** | **Concepts** | **Main Concepts** |
| --- | --- | --- | --- |
| Plan and develop the program | Women experienced lack of information and screening for breast cancer  Awareness and knowledge on breast cancer and early detection measures were affected by limited coverage, clinicians work load, health system structure, education and literacy levels of women, attitude towards the disease and financial challenges.  Attitude towards the disease, priority to social responsibilities, influence by social network negatively influenced breast screening practices and time of seeking care for a breast symptom  The support and engagement by HP with vision and will have impact on breast health services and its implementation  Knowing about the existence of breast health services will allow time for its up- take as felt by women  The availability of HPs who have the skills and knowledge to deliver and implement breast health services effectively with available resources | Awareness of existing deficiencies  Awareness of the need for breast service program  Vision (what, where, when, why, by who, and for whom)  Activities to be carried out  Program agents  Champions for the program | **Initiate and sustain BC prevention and early detection program**  Plan for the program  Management commitment  Readiness of HPs  Vision for the program  Implementation plan  Uptake by patient, family and MC  Availability of resources  Develop program structure |
| Interaction with stakeholders | The HPs perceived that women’s health beliefs about breast cancer are shaped by existing perceptions about the disease within the family and community    The HPs perceived women and their communities as playing a vital role in the implementation of BC prevention and early detection program  Effective communication of BC prevention and early detection program influences engagement of the stakeholders  HPs and women perceived interaction as the driving-force for effective implementation of breast prevention and early detection program | Women’s health beliefs about BC  Existing family and community perception about the disease  Patient and family awareness  MCs awareness  Community awareness  Communication of BC prevention and early detection program by family and MCs  Involvement of the health team and women as multidisciplinary stakeholders | **Collaboration between health professionals, patients, families, MCs and communities**  Interaction between HPs and patients, families and MCs  Engagement sessions with women  Awareness sessions with women  Multidisciplinary team |
| Environment Resource and support | Lack of breast awareness and centers for women.  Breast cancer protocol and guideline were not in existence to guide breast health care at the national and facility level  Commitment of policy makers towards breast cancer influenced the provision of BC prevention and early detection program  HP perceived high clinician-patient ratio affects effective implementation of BC prevention and early detection program  The context for the implementation of the program was perceived as having influence on its implementation  Lack of adequate staffs, lack of space and materials (breast cancer leaflets) affects the implementation of the program  HPs experienced lack of support and recognition, resulting in reservation about the effectiveness of introduced program | Physical space  Program’s policy  Working load  Staffing  Materials  Equipment’s | **Conducive environment of the health care facility and needed resources**  Availability of space  Increase staff strength  BC and BSE leaflets/broachers  Management commitment and support  Accept and support BC innovation  Policy for effect implementation  Attitude  Self-motivated HPs |
| Capacity building | HPs perceived the lack of women and clinician’s knowledge on BC, competencies in counselling, breast examination techniques and attitude towards the disease as influencing on breast cancer timely presentation  The deficiencies in breast examination techniques by both women and clinicians was perceived as impacting on BC and early detection  The knowledge of women and HPs on BC prevention and early detection was perceived as relevant | Training of HPs (seminar, in-service training)  Education of HPs on BC, its prevention and early detection measures  Train HPs on counselling  Training of HPs on breast examination techniques | **Action**  HPs advocacy  Breast health services through clinical practice |
| Contextual need | The program should include contextual health education, breast screening, and prevention of breast cancer and early detection services for patient’ family and micro-community. | Education sessions with family and MCs  Teach family and MCs BSE  Offer CBE to family and MC  Counsel Family and MC before and after CBE  Encourage family and MC to do monthly BSE  Encourage period CBE  Urge women to report with any suspicion | **Services**  Breast cancer health education  Tutorials on BSE  CBE  Counseling  Follow- up care |
| Communication approach and expansion of the program | HPs perceived communication plan to the community as having influence on the uptake of the program due to the social stigma attached to the disease  Awareness of other HPs, women and their communities about the program and its implementation was perceived as impediment to the BC prevention and early detection program  Breast health services should be extended into the community through family and micro-community | Effective communication  Productive communication to the community  Timely communication  Dissemination of program  Extend invitation into the program to community through family and MC | **Diffusing innovation into the community through agents**  Engagement with patient, family and MC  Family and MC involvement with information disseminate within the community  Invite community members  Feedback |
